# The Systemic Lupus Erythematosus Infection Predictive Index (LIPI): A Clinical-Immunological Tool to Predict Infections in Lupus Patients

**DOI:** 10.3389/fimmu.2018.03144

**Published:** 2019-01-14

**Authors:** Jiram Torres-Ruiz, Nancy R. Mejía-Domínguez, Alejandro Zentella-Dehesa, Alfredo Ponce-de-León, Sandra Rubí Morales-Padilla, Ricardo Vázquez-Rodríguez, Mario René Alvarado-Lara, Roberto Adrián Reyna-de-la-Garza, Miguel Tapia-Rodríguez, Guillermo Juárez-Vega, Javier Merayo-Chalico, Ana Barrera-Vargas, Jorge C. Alcocer-Varela, Diana Gómez-Martín

**Affiliations:** ^1^Department of Immunology and Rheumatology, Instituto Nacional de Ciencias Médicas y Nutrición Salvador Zubirán, Mexico City, Mexico; ^2^Emergency Medicine Department, Instituto Nacional de Ciencias Médicas y Nutrición Salvador Zubirán, Mexico City, Mexico; ^3^Bioinformatics, Biostatistics and Computational Biology Unit, Red de Apoyo a la Investigación, Coordinación de la Investigación Científica, Universidad Nacional Autónoma de México, Mexico City, Mexico; ^4^Department of Genomic Medicine and Environmental Toxicology, Instituto de Investigaciones Biomédicas, Universidad Nacional Autónoma de México, Mexico City, Mexico; ^5^Department of Infectology and Microbiology, Instituto Nacional de Ciencias Médicas y Nutrición Salvador Zubirán, Mexico City, Mexico; ^6^Microscopy Unit, Instituto de Investigaciones Biomédicas, Universidad Nacional Autónoma de México, Mexico City, Mexico; ^7^Flow Cytometry Unit, Red de Apoyo a la Investigación, Coordinación de Investigación Científica, Universidad Nacional Autónoma de México, Mexico City, Mexico

**Keywords:** systemic lupus erythematosus, infection predictive index, TLR2, Th17, cyclophosphamide

## Abstract

Among autoimmune diseases, systemic lupus erythematosus (SLE) patients have a unique predisposition to develop infections, which represents one of their main causes of morbidity and mortality. Many infections occur at disease diagnosis in the absence of immunosuppressive therapy, suggesting that the immunological abnormalities in SLE patients might be fundamental for the development of this complication. The aim of this study was to address the main clinical and immunological features associated with the development of infection and to create and validate a compound clinical-immunological infection predictive index in a cohort of SLE patients. We included 55 SLE patients with < 5 years since diagnosis. The clinical and immunological features were evaluated periodically and patients were followed-up during 1 year, searching for the development of infection. Immunophenotyping was performed by multiparametric flow cytometry and neutrophil extracellular traps (NETs) were assessed by confocal microscopy. Eighteen patients (32.7%) presented 19 infectious events, 5 (26.3%) were severe. For the construction of the index, we performed a logistic regression analysis and the cutoff points were determined with ROC curves. Increased numbers of peripheral Th17 cells, B cell lymphopenia, and lower TLR2 expression in monocytes, as well as the use of cyclophosphamide were the major risk factors for the development of infection and thus were included in the index. Besides, patients that developed infection were characterized by increased numbers of low-density granulocytes (LDGs) and higher expression of LL-37 in NETs upon infection. Finally, we validated the index retrospectively in a nested case-control study. A score >1.5 points was able to predict infection in the following year (AUC = 0.97; LR– = 0.001, specificity 100%, *P* = 0.0003). Our index encompasses novel immunological features able to prospectively predict the risk of infection in SLE patients.

## Introduction

Systemic lupus erythematosus (SLE) is the prototypic multi-organic autoimmune disease, and it implies high morbidity and early death in young and productive people (1). The immunopathology of SLE encompasses multiple innate and adaptive immunologic alterations, including hypocomplementemia, higher levels of TNF-α, IL-4, IL-6, IL-10, as well as type I and II interferons, with a consequent skewing toward a Th17 response, persistent B-cell activation with sustained auto-antibody secretion and a deficient regulatory T cell profile (2).

Patients with SLE also have higher amounts of low-density granulocytes (LDGs), which infiltrate tissues, secrete pro-inflammatory cytokines and spontaneously produce neutrophil extracellular traps (NETs) (3). Aside from the role of NETosis in the SLE pathogenesis, the NETs are an innate defense mechanism since they contain antimicrobial proteins including LL-37 (3).

Moreover, previous studies have shown that patients with SLE have expansion of the pro-inflammatory intermediate monocytes in peripheral blood (4). Toll-like receptor 2 (TLR2) is one of the mainly expressed pattern recognition receptor in monocytes, has diverse ligands (5), and it is pivotal in the SLE pathophysiology since it promotes an interferogenic response and has been shown to augment disease activity in an animal model of SLE through the recognition of the bacterial curli (5).

Infections are one of the main causes of hospital admissions in patients with SLE (6), and they represent one of the three major causes of death, along with renal and cardiovascular diseases (7). In patients with SLE, infections are traditionally considered a complication of immunosuppressive therapy, as was confirmed in a recent retrospective cohort study (8) and therefore, most of the infection prophylactic measures in SLE are directed at patients using immunosuppressive therapy (9). Nevertheless, 25.9% of severe infections in patients with SLE occur at diagnosis in the absence of immunosuppressive therapy (10) and previous studies have described disease activity and number of flares as independent risk factors for infections (11), which suggests an inherent infection predisposition that may be dependent upon the immunologic abnormalities that characterize the disease (2).

Currently, there is not an available tool to identify SLE patients at high risk of infection, regardless of steroid treatment in the routine clinical scenario. Thus, the development of an infection prediction index that includes the clinical and immunological features of SLE patients is crucial to identify a group at higher risk to develop this complication. The aim of this study was to prospectively create and validate a compound clinical-immunological index that was useful to predict the development of infections in SLE patients in the following year.

## Methods

### Construction of a Cohort of SLE Patients

**Figure 1:**
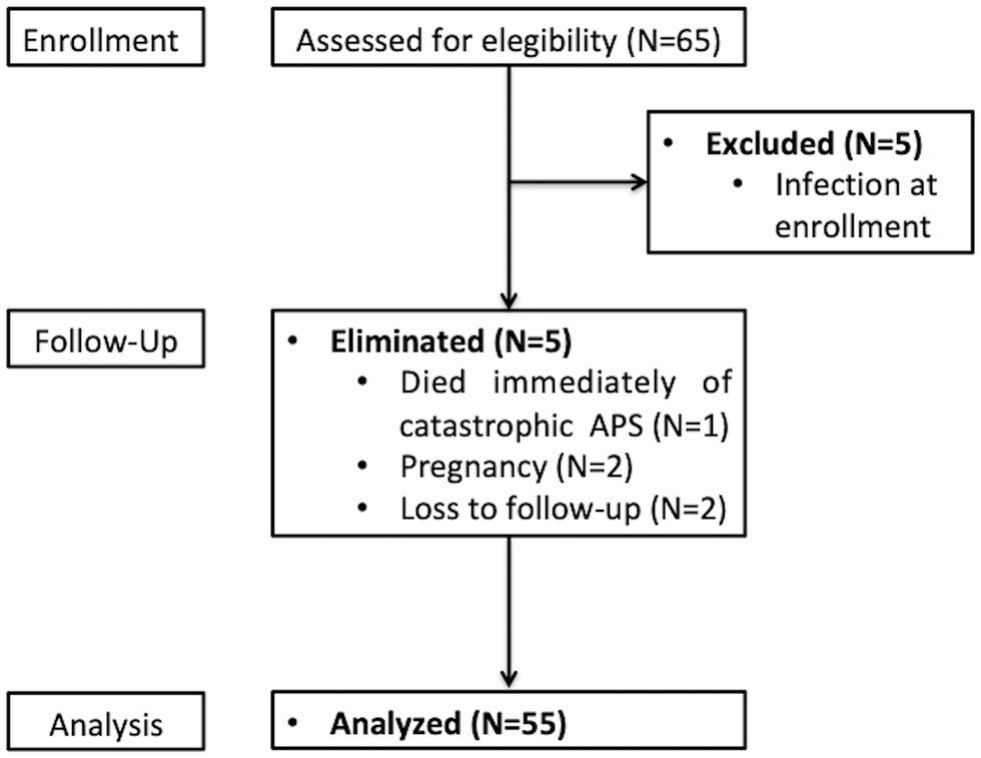
Flowchart of patient enrollment, follow-up, and analysis.

From 2015 to 2017, we performed a prospective cohort study in which we recruited 65 consecutive Hispanic adult patients with classified SLE using the American College of Rheumatology (ACR) (12) and/or the Systemic Lupus International Collaborating Clinics (SLICC) criteria (13) with < 5 years since the diagnosis who were followed-up in a tertiary care center in Mexico City. Patients with active infection at the time of recruitment, overlap syndromes (except antiphospholipid syndrome), chronic viral infections, cancer, primary immunodeficiencies, pregnancy, puerperium, end-stage renal disease, and late onset SLE were excluded because of the inherent immunologic alterations in these subjects (14, 15). The study was approved by our institutional Ethics committee in compliance with the Helsinki declaration and all subjects provided their informed consent prior to inclusion. Nine patients were eliminated and one died of catastrophic antiphospholipid syndrome and severe infections immediately after recruitment (Figure 1).

To create an index that was able to predict the development of infection in the following year, 55 patients were followed up during 12 months, looking for the primary outcome that was the development of infection, defined as the presence of characteristic clinical features with response to antibiotic or antiviral treatment, regardless of microbiological isolation. Severe infections were defined as those requiring hospital admission for at least 72 h, intravenous antibiotic treatment or causing death (6, 10). Patients were evaluated at the following time points: baseline, after 1, 3, 6 months and at the time of infection. During all the visits, we registered the laboratory data (including complement levels measured by nephelometry, anti-double stranded DNA (dsDNA) and anti-nucleosome antibodies assessed by ELISA), the type and dose of immunosuppressive therapy and disease activity with the Safety of Estrogens in Lupus National Assessment-Systemic Lupus Erythematosus Disease Activity Index, (SELENA/SLEDAI) (16) and British Isles Lupus Assessment Group (BILAG 2004) (17) scales. At baseline and during infection, we measured anti-Ro/La and anti-Sm antibodies by ELISA. Unless the patient had the previous diagnosis of anti-phospholipid syndrome, we assessed anti-phospholipid antibodies by ELISA and lupus anticoagulant with diluted Russell viper venom time in two occasions, 12 weeks apart.

### Determination of the Immunologic Parameters of SLE Patients

At baseline, in the 3 and 6 months visits and during infection we evaluated the following immunological features of patients with SLE:

#### Immunophenotyping by Multiparametric Flow Cytometry

Peripheral blood mononuclear cells (PBMCs) were isolated by density gradients after centrifugation with Lymphoprep (Stemcell Technologies, Vancouver, Canada). PBMCs were re-suspended in RPMI with phenol red (Thermo Fisher scientific), washed twice with 5% FBS (fetal bovine serum) in PBS and stained with the following fluorescent labeled-antibodies: CD3, CD8, CD4, CD25, CD127, CD14, CD16, CD10, CD15, CD282 (TLR2), CD56, CD335 (NKp46) (BD-Biosciences, Franklin Lakes, New Jersey, USA). The peripheral cell subsets were defined as following: CD8 T cells (CD3+, CD8+), CD4 T cells (CD3+, CD4+), B cells (CD3−, CD19+), NK cells [CD3−, CD335+ (NKp46), CD56+], regulatory T cells (CD4+, CD25^hi^ CD127^lo/−^), LDG (CD10+, CD14−, CD15+), classical monocytes (CD14+, CD16−), intermediate monocytes (CD14^hi^, CD16+) and non-classical monocytes (CD14^lo^, CD16+). Additionally, we measured the percentage of total and intermediate monocytes that were positive for TLR2 and its expression in those cell populations by mean fluorescence intensity (MFI) using the appropriate mouse IgG kappa-BV421 isotype control (BD-Biosciences, Franklin Lakes, New Jersey, USA).

To assess the percentage of T helper (Th) subsets, PBMCs were stimulated with PMA (phorbol myristate acetate; 50 ng/mL) and ionomycin (1 μg/mL) and treated with monensin during 5 h. The cells were washed twice with 5% FBS in PBS and stained with anti-CD4 (BD-Biosciences, Franklin Lakes, New Jersey, USA). After fixation and permeabilization at 4°C, we stained for intracytoplasmic IFN-γ, IL-4, and IL-17 (BD-Biosciences, Franklin Lakes, New Jersey, USA). All samples were acquired in an LSR FORTESSA flow cytometer (BD-Biosciences, Franklin Lakes, New Jersey, USA) and analyzed with the software Flow-jo V10 (Flow-jo LLC). The absolute number of every cellular subset was calculated taking into account the total number of lymphocytes (CD8, CD4, NK, B cells), monocytes (classical, intermediate, non-classical and TLR2-positive monocytes), and leukocytes (LDGs) of a complete blood cell count taken at the time of the blood draw.

#### Induction and Quantification of NETs and LL-37

After density gradients, conventional neutrophils were isolated with dextran sedimentation. We quantified the spontaneous NET formation (without stimuli) and LPS-induced NETosis with 1 μg/mL *E. coli* O111:B4 LPS (Sigma Aldrich, St. Louis Missouri, USA) by fluorescence spectrometry and indirect immunofluorescence. Briefly, neutrophils were incubated in RPMI without phenol red (Thermo Fisher scientific), 1% FBS, and 1% 10 mM HEPES at 37°C during 1.5 h in dark 96 wells plates with 0.2 μM SYTOX green (Thermo Fisher Scientific, Waltham, Massachusetts USA). The experiments were repeated by quadruplicate for the spontaneous and LPS-induced NETosis and measured with a Biotek Sinergy HT Spectrofluorometer (Biotek, Winooski, VA, USA). Additionally, spontaneous and LPS-induced NETosis was quantified by confocal microscopy. Conventional neutrophils were seeded in 0.01% poly-L-Lysine (Sigma-Aldrich, Germany) coated coverslips at 37°C during 1.5 h. Cells were fixed with 4% paraformaldehyde (Santa Cruz Biotechnology, USA) at 4°C during 24 h. After blocking with 0.02% gelatin from porcine skin, we performed indirect immunofluorescence using the following primary antibodies: rabbit anti-human neutrophil elastase 1: 500 (Abcam, Cambridge, United Kingdom) and mouse anti-human LL-37 1: 500 (Santa Cruz Biotechnology, Dallas, Texas, USA) (18). The following secondary antibodies were used: Donkey anti-rabbit Alexa Fluor 555 1: 500 (Thermo Fisher, Waltham, Massachusetts, USA) and donkey anti-mouse DyLight 488 1: 250 (Thermo Fisher, Waltham, Massachusetts, USA). The primary and secondary antibodies were diluted in 0.02% gelatin from porcine skin. Chromatin was stained with 1: 1,000 Hoechst 33342 (Thermo Fisher, Waltham, Massachusetts, USA) and coverslips were mounted on slides with ProLong® Gold Antifade Mountant (Thermo Fisher, Waltham, Massachusetts, USA). The samples were acquired in an Eclipse Ti-E Nikon confocal microscope (Minato, Tokyo, Japan). The amount of NETs was quantified as the mean number of fibrillar structures in which chromatin co-localized with neutrophil elastase divided by the number of cells and multiplied by 100 in six 40X fields per experimental condition (spontaneous and LPS-induced) (18). The expression of LL-37 was quantified as the MFI of DyLight Alexa fluor 488 in every NET in six 40X fields per experimental condition. The quantification of NETs was done in a blinded fashion. The images were analyzed with the software Fiji (NIH). The baseline immunological parameters of patients with SLE were compared with 20 age and sex-matched healthy controls (18).

### Statistic Analysis

Quantitative variables were expressed as medians with interquartile ranges (IQR). Association between nominal variables was assessed with the Chi-square test. To encompass the variability of disease activity and immunosuppressive therapy throughout time, we calculated the adjusted mean SLEDAI score (19) and performed a repeated measure analysis for the presence or absence of immunosuppressive therapy and for the prednisone dose, that was categorized as low (≤7.5 mg/d), medium (>7.5 mg-30 mg/d), and high (>30 mg/d) (20) in each visit. To compare the paired medians of the parameters at baseline and during the infectious event we used the Wilcoxon test. Since all patients presented the primary outcome at different time-points, we performed a Cox proportional hazard model with the time until infection as the primary outcome. None of the parameters were able to predict the time until the infectious event. Therefore, to develop the systemic lupus erythematosus (SLE) infection predictive index (LIPI), we performed a univariate and multivariate logistic regression analysis in which we used the baseline measurements of each member of the cohort. Also, we carried out a cohort-nested case-control analysis using the measurements taken 1–3 months previous to the development of infection and comparing them with those taken in patients whom did not developed infection with the same follow-up time. Relative risks (RR) with 95% confidence intervals (95% CI) were calculated in the two data sets.

The variables that showed a statistically significant association were taken to create different predictive models of infection. The explanatory model with the lowest Akaike Information Criterion (AIC) value was chosen (21). Afterwards, from this explanatory model, we selected the variables with significant RR to be included in the index. Also, we estimated the cut-off points for each of these variables with the Receiver Operating Characteristic (ROC) curves based on the smallest sum of squares of 1-sensitivity and 1-specificity. A numerical value was assigned to each of the index variables according to RR value (22, 23), where the highest value (3) corresponded to the highest RR. Finally, we compared different models that included the potential combinations of variables selected for the index based on their area under the curve (AUC), sensitivity and specificity with the baseline data as well as in the cohort nested case-control study, which was done for the retrospective validation. We selected the model with the best performance for infection prediction, measured as the AUC by the ROC test (24). A *P* < 0.05 was considered significant. The statistical analysis was made with the SPSS v21 (IBM Corp. Armonk, NY, USA) and R package (R Core Team) software.

## Results

Seventy-five percent of patients were women. Median time since SLE diagnosis was 13 months (IQR 4-28). The main clinical manifestations at SLE diagnosis were articular (84%), mucocutaneous (81%), renal (50%), hematologic (37%), serositis (37%), constitutional (35%), neurological (6%), and 14% had secondary anti-phospholipid syndrome.

As previously described, when compared to healthy controls, patients with SLE had a diminished count of lymphocytes (25) and regulatory T cells (26) and a higher percentage of LDGs (27), Th1, Th2 and Th17 cells (28). Moreover, patients with SLE had lower absolute number of B cells and a lower expression of TLR2 in total monocytes (Table 1).

**Table 1:** Baseline immunologic characteristics of patients with SLE and healthy controls.

**Parameter**	**SLE median (IQR) *N* = 55**	**Healthy control median (IQR) *N* = 20**	***P***
Age (years)	25 (21–34)	25 (24–25.8)	0.94
**LABORATORY CHARACTERISTICS**			
Leukocytes (×10^9^/L)	5.75 (4.15–7.77)	6.85 (6.10–7.95)	**0.016**
Lymphocytes (×10^6^/L)	1,084 (686–1,659)	2,119 (1,939–3,022)	**<0.001**
Neutrophils (×10^6^/L)	3,724 (2,881–5,451)	3,935 (3,000–4,415)	0.94
Monocytes (×10^6^/L)	450 (288–590)	576 (500–576)	**0.007**
Hemoglobin (gr/dL)	13.7 (11.9–14–7)	15 (15, 16)	**<0.001**
Platelets (×10^9^/L)	257 (191–306)	300 (270–400)	**0.001**
**IMMUNOLOGIC FEATURES**			
CD4+ lymphocytes (×10^6^/L)	247 (79.5–611.25)	881 (770–990)	**<0.001**
CD4+ lymphocytes (%)	39.9 (33.8–48.52)	62.2 (54.2–67)	**<0.001**
CD8+ lymphocytes (×10^6^/L)	212 (82–488)	465 (381–670)	**0.002**
CD8+ Lymphocytes (%)	30.4 (24–36.1)	31.8 (27–38.5)	0.79
B cells (×10^6^/L)	163.5 (55.75–352.25)	327 (177–434)	**0.009**
B cells (%)	36.1 (14.3–36.1)	37.5 (28.3–54.5)	0.47
Th1 (×10^6^/L)	3 (2–9)	3 (1.25–10.5)	0.621
Th1 (%)	0.8 (0.46–4.65)	0.29 (0.2–1.37)	**0.003**
Th2 (×10^6^/L)	4 (2.5–12.5)	5.5 (3–14.25)	0.39
Th2 (%)	1.35 (0.8–2.25)	0.55 (0.39–1.75)	**0.026**
Th17 (×10^6^/L)	4 (2–10.5)	4 (2–7.75)	0.917
Th17 (%)	1.57 (0.5–1.94)	0.4 (0.3–0.65)	**0.001**
Regulatory T cells (×10^6^/L)	3 (0.7–10)	22 (8–29.5)	**<0.001**
Regulatory T cells (%)	0.7 (0.4–1.4)	1.7 (1–3)	**<0.001**
NK cells (×10^6^/L)	47 (8.5–126.5)	202 (114–494)	**<0.001**
NK cells (%)	13 (5.9–26.9)	33 (24.25–52)	**<0.001**
Intermediate monocytes (×10^6^/L)	18 (5–60)	40.5 (16.5–60.5)	0.168
Intermediate monocytes (%)	4.2 (1.8–17)	8.15 (3.2–11.37)	0.279
Non-classical monocytes (×10^6^/L)	5 (1.4–16.15)	8 (2.6–11)	0.416
Non-classical monocytes (%)	2 (0.55–4.1)	2 (1.2–2.1)	0.215
TLR2+ monocytes (×10^6^/L)	365 (164–542)	440 (328–581.25)	0.106
TLR2 monocytes (%)	83 (65.5–87.75)	87.5 (73–92)	0.163
TLR2+ intermediate monocytes (×10^6^/L)	18.5 (5–62.75)	45 (18.2–69.25)	0.120
TLR2 intermediate monocytes (%)	98.3 (96–99.2)	99 (98.12–99)	0.218
TLR2 mean fluorescence intensity in total monocytes	9,567 (5,552–13,848)	16,890 (11,124–22,256)	**0.005**
TLR2 mean fluorescence intensity in intermediate monocytes	16,293 (7,583–21,263)	19,717 (13,063–25,963)	0.113
Low density granulocytes (×10^6^/L)	43 (19–110)	16.5 (6.2–19)	**<0.001**
Low density granulocytes (%)	10.3 (5.6–30.77)	1.9 (1.5–2.95)	**<0.001**
Spontaneous NETs^a^ (Sytox green fluorescence intensity)	2,133 (1,226–2,695)	1,744 (977–2,448)	0.271
LPS-induced NETs (Sytox green fluorescence intensity)	1,926 (1,163–2,785)	1,759 (1,188–2,490)	0.432
Spontaneous NETs (NETs/number of cells)	33 (7.5–125)	21 (10–50)	0.282
LPS-induced NETs (NETs/number of cells)	35 (7–99)	2 (0–17)	**<0.001**
Mean fluorescence intensity of LL-37 in spontaneous NETs	66.15 (27.9–97.87)	36.64 (0–113.75)	0.072
Mean fluorescence intensity of LL-37 in LPS-induced NETs	78 (32.4–123.9)	171 (78.5–211)	**0.001**

a *Neutrophil extracellular traps. The statistically significant P values are represented in bold*.

During 12 months of follow-up, 18 patients (32%) developed 19 infectious events in a median time of 21.5 weeks (IQR 4-24), 5 (26.3%) were severe. The main types of infections were community-acquired pneumonia (23%), upper respiratory tract (23%) and urinary tract infections (17%), herpes zoster virus (17%), gastroenteritis (11%), and cellulitis (5%). During the study period, 6 (10.9%) patients were receiving cyclophosphamide, 4 (66.6%) of them had an infection.

**Figure 2:**
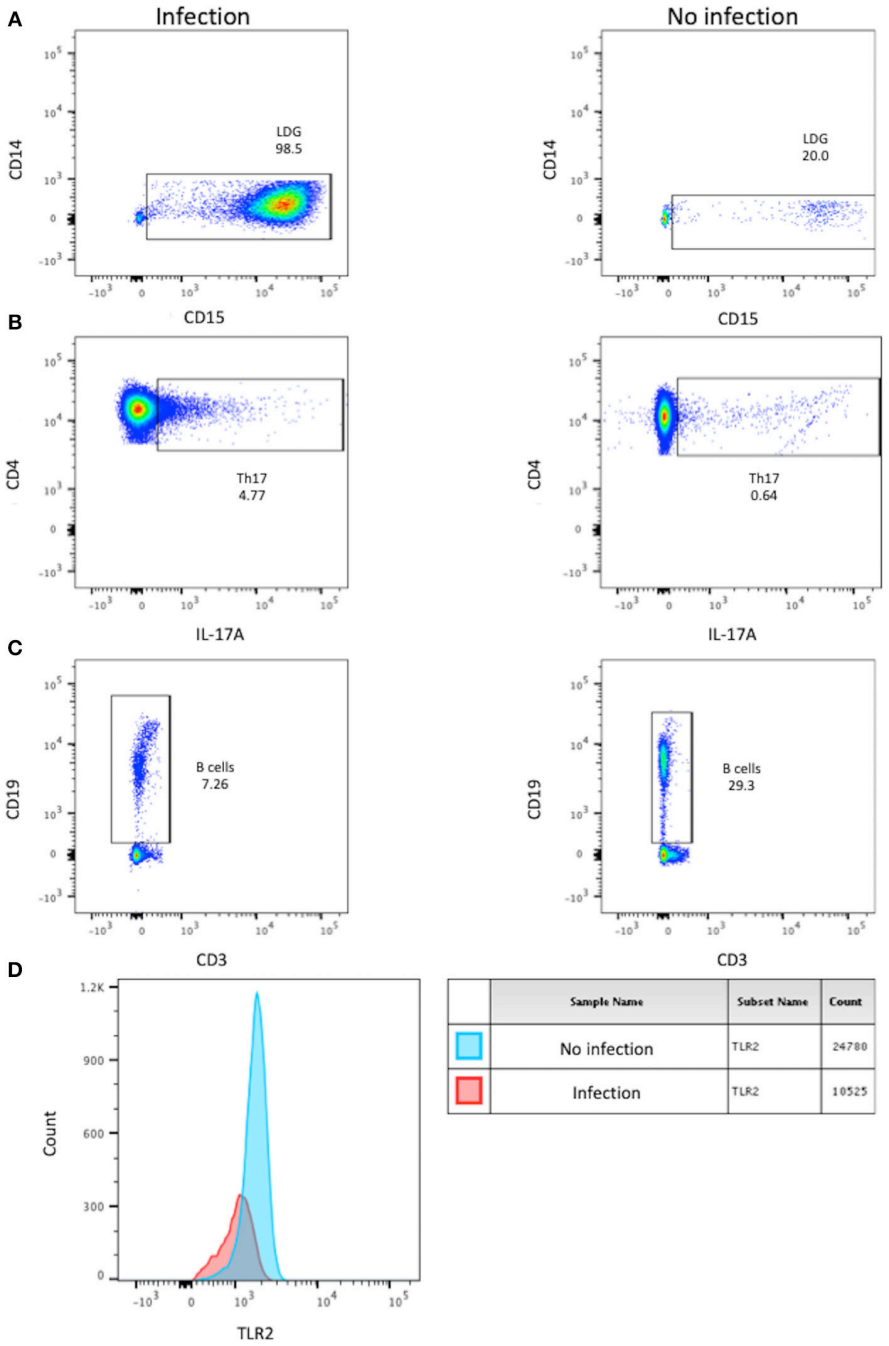
Representative dot plots of LDG **(A)**, Th17 lymphocytes **(B)**, B cells **(C)** that were significantly different at baseline in patients with and without the prospective development of infection. Representative histogram of the proportion of total TLR2+ monocytes and the expression of TLR2 between patients with and without the prospective development of infection **(D)**.

As shown in Table 2 and Figure 2, patients that developed infection during the follow-up period had higher amounts of anti-dsDNA antibodies, LDGs, Th17 cells, and diminished absolute number of B cells at baseline. We found no association between the vaccination status, tobacco use and prophylactic antibiotic administration with the development of infection (data not shown).

**Table 2:** Baseline characteristics of patients with SLE according to the prospective development of infection.

**Parameter**	**Infection median (IQR) N = 18**	**No infection median (IQR) N = 37**	**P**
**DEMOGRAPHIC FEATURES**			
Female (%)	17 (68)	27 (69.2)
Age (years)	25 (20–30)	25 (21–37)	0.54
Time since SLE diagnosis (months)	13 (2.5–28)	12.5 (4–30.25)	0.52
**LABORATORY CHARACTERISTICS**			
Leukocytes (×10^9^/L)	6.05 (4.25–10.15)	5.3 (3.8–6.83)	0.13
Lymphocytes (×10^6^/L)	989 (557–1,697)	1,117 (720–1,655)	0.8
Neutrophils (×10^6^/L)	4,437 (3,148–7,966)	3,571 (2,545–5,063)	0.08
Monocytes (×10^6^/L)	493 (364–614)	357 (255–576)	0.16
Hemoglobin (gr/dL)	14.3 (11.9–14.7)	13.5 (11.9–14.5)	0.54
Platelets (×10^9^/L)	270 (190–310)	241 (190–301)	0.54
Creatinine (mg/dL)	0.66 (0.58–0.9)	0.7 (0.58–0.8)	0.88
Blood urea nitrogen (mg/dL)	14.9 (9.8–23.4)	12.8 (10.2–16.7)	0.48
24 h urinary protein (gr/d)	0.31 (0.13–3.35)	0.3 (0.1–1.2)	0.35
Protein/creatinine index (mg/mg)	0.41 (0.1–2.9)	0.13 (0.07–1)	0.18
C-reactive protein (mg/L)	0.32 (0.09–0.67)	0.25 (0.11–1)	0.9
Erythrocyte sedimentation rate (mm/h)	16 (6.5–31.5)	12 (4.2–19)	0.12
**CONVENTIONAL IMMUNOLOGICAL FEATURES**			
C3 (mg/dL)	98 (61.5–111.5)	96 (71.25–113.5)	0.75
C4 (mg/dL)	13 (8–21.5)	11 (8–20)	0.98
Anti nucleosome antibodies (IU/L)	302 (115–507)	235 (80.1–422)	0.34
Anti dsDNA^a^ antibodies (IU^b^/L)	130 (79–793)	78.45 (27.1–223)	**0.039**
Anti Ro antibodies (IU/L)	35.3 (6.9–546.2)	9 (5.4–280)	0.15
Anti La antibodies (IU/L)	5 (3.9–6.1)	4.1 (3.1–6)	0.2
Anti Smith antibodies (IU/L)	10.6 (7.2–82.7)	7.6 (6.8–57.8)	0.44
Anti cardiolipin IgG (IU/L)	6.6 (5–8.9)	6.1 (4.9–8.5)	0.6
Anti β2-GP^c^ IgG (IU/L)	4.1 (3.8–4.3)	4 (3.5–4.7)	0.6
Anti cardiolipin IgM (IU/L)	13.1 (8.3–19.6)	8.1 (6.7–12.7)	0.058
Anti β2-GPI IgM (IU/L)	6.1 (4.6–10.97)	4.8 (4.2–7.2)	0.085
**DISEASE ACTIVITY AND ACCRUAL DAMAGE SCALES**			
SELENA/SLEDAI^d^	12 (0–16)	8 (4–12)	0.71
BILAG^e^	12 (0–18)	4.5 (0–12)	0.26
SLICC/SDI^f^	0 (0–0)	0 (0–0)	0.68
**NOVEL IMMUNOLOGICAL FEATURES**			
CD4+ lymphocytes (×10^6^/L)	217 (22–703)	254 (154–500)	0.43
CD4+ lymphocytes (%)	42.4 (26.7–49.3)	39 (34–49)	0.98
CD8+ lymphocytes (×10^6^/L)	218 (31–439)	170 (82–415)	0.7
CD8+ lymphocytes (%)	31.7 (26.5–43.1)	29.7 (23.1–36)	0.39
B cells (×10^6^/L)	88 (16–254)	253 (97–381)	**0.028**
B cells (%)	34 (12.2–56.5)	38.2 (16.3–71.8)	0.4
Th1 (×10^6^/L)	4 (2–30)	3 (2–6)	0.4
Th1 (%)	0.8 (0.26–11.8)	0.8 (0.5–2.6)	0.7
Th2 (×10^6^/L)	6 (3–15)	3.75 (1.75–6.25)	0.18
Th2 (%)	1.3 (0.55–3.6)	1.4 (0.8–2)	0.67
Th17 (×10^6^/L)	11 (4–43)	3 (2–4)	**0.007**
Th17 (%)	1.8 (1.4–2.52)	0.9 (0.3–1.6)	**0.01**
Regulatory T cells (×10^6^/L)	1 (0.2–10)	3.5 (1–8.5)	0.26
Regulatory T cells (%)	0.7 (0.41–1)	0.85 (0.4–1.47)	0.36
NK cells (×10^6^/L)	126 (19–160)	27 (6–82)	0.078
NK cells (%)	14.3 (9.3–36.2)	10.6 (4–44)	0.13
Intermediate monocytes (×10^6^/L)	58.5 (3.75–168.75)	16 (5–44)	0.12
Intermediate monocytes (%)	7.8 (2–20)	3.8 (1.7–11.2)	0.1
Non-classical monocytes (×10^6^/L)	6 (2–27)	4.6 (1–15.4)	0.26
Non-classical monocytes (%)	1.9 (0.5–5.6)	0.76 (0.52–3)	0.39
TLR2+ monocytes (×10^6^/L)	433 (230–552)	308 (162–514)	0.33
TLR2+ monocytes (%)	87 (73–92)	80.9 (62.5–84.9)	0.16
TLR2+ intermediate monocytes (×10^6^/L)	71 (6–167)	15 (5–44)	0.06
TLR2 intermediate monocytes (%)	98.3 (91.4–99)	98.3 (96–99.5)	0.45
TLR2 mean fluorescence intensity in total monocytes	11,553 (1,436–18,625)	9,538 (5,976–12,074)	0.51
TLR2 mean fluorescence intensity in intermediate monocytes	18,076 (1,674–21,727)	14,584 (8,779–19,836)	0.52
Low density granulocytes (×10^6^/L)	105 (34–331)	32 (14–77)	**0.006**
Low density granulocytes (%)	17.7 (8.7–37)	8 (4.2–26.5)	0.09
Spontaneous NETs^g^ (Sytox green fluorescence intensity)	1,610 (1,071–2,581)	2,192 (1,258–2,800)	0.2
LPS-induced NETs (Sytox green fluorescence intensity)	1,750 (1,044–2,261)	2,328 (1,324–3,358)	0.06
Spontaneous NETs (NETs/number of cells)	33 (12–78)	47 (6–100)	0.72
LPS-induced NETs (NETs/number of cells)	41 (5–110)	33 (8–142)	0.6
Mean fluorescence intensity of LL-37 in spontaneous NETs	77 (28–135)	64.85 (27.9–93)	0.48
Mean fluorescence intensity of LL-37 in LPS-induced NETs	88 (57–161)	77 (27.22–118.42)	0.3
**TREATMENT**			
Prednisone dose (mg/d)	22.5 (10–38.75)	15 (5–110)	0.2
Azathioprine dose (mg/d)	87.5 (50–105.25)	100 (50–143)	0.47
Mofetil mycophenolate dose (gr/d)	1.75 (1–2.12)	2 (1.5–2.8)	0.23
Cyclophosphamide dose (gr/month)	1 (0.88–1)	ND	ND

a *Double stranded deoxyribonucleic acid*.

b *International Units*.

c *Beta2 glycoprotein I*.

d Safety of Estrogens in Lupus National Assessment-Systemic Lupus Erythematosus Disease Activity Index

e *British Isles Lupus Assessment Group*.

f *Systemic Lupus International Collaborating Clinics/ACR damage index*.

g *Neutrophil extracellular traps*.

*The statistically significant P values are represented in bold*.

**Figure 3:**
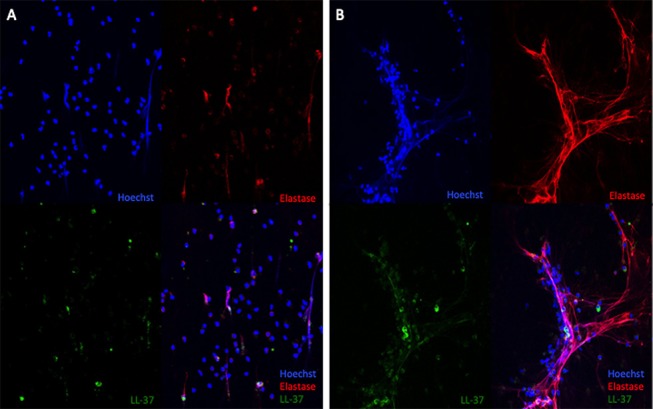
LL37 expression in LPS-induced NETs of a representative patient at baseline **(A)** and during the infectious process **(B)**. 40X.

In comparison to their baseline immunological parameters, there was a higher expression of LL-37 in LPS induced NETs (Figure 3) and a higher amount of LDGs during the infectious events (Table 3).

**Table 3:** Immunologic parameters that were significantly different at baseline and during the infectious event with the Wilcoxon test in patients that developed the outcome (infection).

**Parameter**	**Baseline median (IQR) *N* = 18**	**Infection median (IQR) *N* = 18**	***P***
Anti Smith antibodies (IU/L)	7.8 (6.9–69.3)	9.6 (7.2–24.9)	**0.046**
Th2 (×10^6^/L)	5 (3–13)	2.3 (1.5–14)	**0.028**
Low density granulocytes (×10^6^/L)	26 (14–81)	34.5 (19.4–78.7)	**0.012**
Low density granulocytes (%)	6.49 (2.2–18.8)	10 (4.8–19.6)	**0.016**
Mean fluorescence intensity of LL-37 in LPS-induced NETs^a^	101 (58.2–160.75)	134 (72.5–297)	**0.013**

a *Neutrophil extracellular traps. The statistically significant P values are represented in bold*.

At the time of infection, 10 (55.5%) patients presented an SLE flare. Nevertheless, there were not statistically significant differences in the immunological parameters in comparison to patients who did not flare.

Univariate and multivariate logistic regression analyses were performed to address the association of infection with diverse clinical and immunological parameters at baseline Table 4 and in the cohort-nested case-control study Table 5. The variables associated with infection in multivariate analyses included anti dsDNA antibodies, absolute number of B cells and LDGs and the expression of TLR2 in total monocytes. Nonetheless, as described in Methods, the variables selected for the LIPI were those that were able to predict infection based on the ROC analyses and include the following: the use of cyclophosphamide in the repeated measure analysis, the absolute number of B and Th17 cells and the MFI of TLR2 in total monocytes. The cutoff points for the index were calculated with ROC curves as described in methods and are depicted in Table 6. After the cutoff points were determined, a score was assigned for each variable according to the RR Table 6 as described in methods, and we tested different variable combinations to assess their predictive capability comparing the AUC with the ROC test Table 7.

**Table 4:** Univariate and multivariate analysis to predict infection using the baseline measurements.

	**Univariate analysis**			**Multivariate analysis**		
**Variable**	**RR**^**a**^	**95% CI**^**b**^	***P***	**RR**	**95% CI**	***P***
Anti dsDNA^c^ antibodies (IU^d^/L)	1.0021	1.0006–1.0039	**<0.001**	1.0035	1.0011–1.0073	**<0.001**
Low density granulocytes (×10^6^/L)	1.0057	1.00005–1.0124	**0.04**			
Th17 (×10^6^/L)	1.964	1.117–5.362	**0.001**			
LPS-induced NETs (Sytox green fluorescence intensity)	0.00453	0.0016–0.7770	**0.03**			
Mean fluorescence intensity of TLR2 in intermediate monocytes	0.99993	0.9998–1.00001	0.09			
Mean fluorescence intensity of TLR2 in total monocytes	0.9998	0.9997–1.000006	0.06			

a *Relative risk*.

b *95% confidence interval*.

c *Double stranded deoxyribonucleic acid*.

d *International Units*.

*The statistically significant P values are represented in bold*.

**Table 5:** Univariate and multivariate analysis to predict infection in the cohort-nested case-control study.

	**Univariate**			**Multivariate**		
**Variable**	**RR**^**a**^	**95% CI**^**b**^	***P***	**RR**	**95% CI**	***P***
B cells (×10^6^/L)	0.9959	0.9893–0.9998	**0.038**	0.9827	0.9557–0.9980	**0.01**
Intermediate monocytes (×10^6^/L)	1.0124	1.0008–1.0342	**0.02**			
Low density granulocytes (×10^6^/L)	1.0173	1.0041–1.0398	**<0.001**	1.0457	1.0163–1.1079	**<0.001**
Blood Urea Nitrogen (mg/dL)	1.1224	1.0120–1.3186	0.09			
NK cells (%)	1.0496	0.9997–1.1193	0.08			
Mean fluorescence intensity of TLR2 in total monocytes				0.9997	0.9991–1.00002	0.0720

a *Relative risk*.

b *95% confidence interval*.

*The statistically significant P values are represented in bold*.

**Table 6:** Cutoff points and score assignment according to the relative risk of the variables included in the systemic lupus erythematosus infection predictive index (LIPI).

**Parameter**	**Infection**	**No infection**	**RR**^**a**^	**95% CI**^**b**^	***P***	**Score**	**Cutoff point**
CYC^c^ use	Yes	No	3.678	1.486–9.102	**0.004**	+3	Yes
Th17 (×10^6^/L)	11 (6–43)	3 (2–4)	1.964	1.117–5.362	**0.001**	+2	>8
B cells (×10^6^/L)	47 (14.75–105)	157 (68–256)	0.995	0.989–0.999	**0.038**	+1	< 60.5
TLR2 mean fluorescence intensity in total monocytes	1,640 (1,362–8,292)	8,396 (1,348–13,012)	0.99	0.99–1	0.06	+1	< 1,364

a *Relative risk*.

b *95% confidence interval*.

c *Cyclophosphamide*.

*The statistically significant P values are represented in bold*.

**Table 7:** Comparison of different explanatory models for infection using the baseline and the cohort-nested case-control study data.

	**Cutoff point**	**LR**^**a**^**+**	**LR–**	**AUC**^**b**^ **(95% CI**^**c**^**)**	**Sensitivity (95% CI)**	**Specificity (95% CI)**	***P***
**BASELINE DATA**							
CYC^d^ use	Yes	1.2	0	0.58 (0.43–0.73)	100 (90.51–100)	16.67 (4.73–37.38)	0.27
Th17	>8	**1.63**	0	**0.75 (0.56–0.85)**	**100 (76.84–100)**	**38.71 (21.85–57.81)**	**0.027**
B cells	< 60.5	1.1	0.57	0.51 (0.36–0.65)	88 (68.7–97.45)	21.62 (9.82–38.21)	0.87
MFI^e^ TLR2 in total monocytes	< 1,364	**1.04**	1	**0.67 (0.53–0.81)**	**15.38 (4.3–34.87)**	**85.29 (68.94–95.05)**	**0.019**
CYC+Th17	**>0.5**	**5.5**	**0**	**0.90 (0.76–1.05)**	**100 (75.29–100)**	**81.82 (48.22–97.72)**	**0.0007**
CYC+B-cells+MFI TLR2 in total monocytes	**>0.5**	1.68	0.52	0.70 (0.5–0.89)	69.2 (38.5–90.9)	58.8 (32.9–81.5)	0.06
LIPI^f^ (CYC+Th17+B-cells+MFI TLR2 in total monocytes)	**>1.5**		**0.001**	**0.97 (0.91–1.038)**	**90 (55.5–99.75)**	**100 (69.15–100)**	**0.0003**
**COHORT-NESTED CASE CONTROL ANALYSIS**							
CYC use	Yes	1.11	0	0.55 (0.36–0.73)	100 (82.35–100)	10.53 (1.3–33.14)	0.57
Th17	>8	1.48	**0.3**	0.51 (0.26–0.77)	88.89 (65.29–98.62)	40 (12.16–73.76)	0.86
B cells	< 60.5	**1.8**	**0.39**	**0.77 (0.61–0.93)**	**78.95 (54.43–93.95)**	**56.25 (29.88–80.25)**	**0.0051**
MFI TLR2 in total monocytes	< 1,364	0.95	1	0.59 (0.39–0.78)	73.68 (48.8–90.85)	23.53 (6.8–49.9)	0.34
CYC+Th17	>0.5	5.4	0.45	0.75 (0.54–0.96)	60 (26.2–87.8)	88.8 (65.2–98.6)	0.027
CYC+B-cells+TLR2 in total monocytes	>0.5	2.89	0.53	0.71 (0.51–0.91)	57.8 (73.9–79.7)	80 (44.3–97.4)	0.06
LIPI^e^ (CYC+B-cells+TLR2 in total monocytes+Th17)	>1.5	5.4	0.45	0.76 (0.55–0.96)	60 (26.4–87.8)	88.8 (65.2–98.62)	0.024

a *Likelihood ratio*.

b *Area under the curve*.

c *95% confidence interval*.

d *Cyclophosphamide*.

e *Mean fluorescence intensity*.

f *Lupus Infection Predictive Index*.

*The statistically significant P values are represented in bold*.

**Figure 4:**
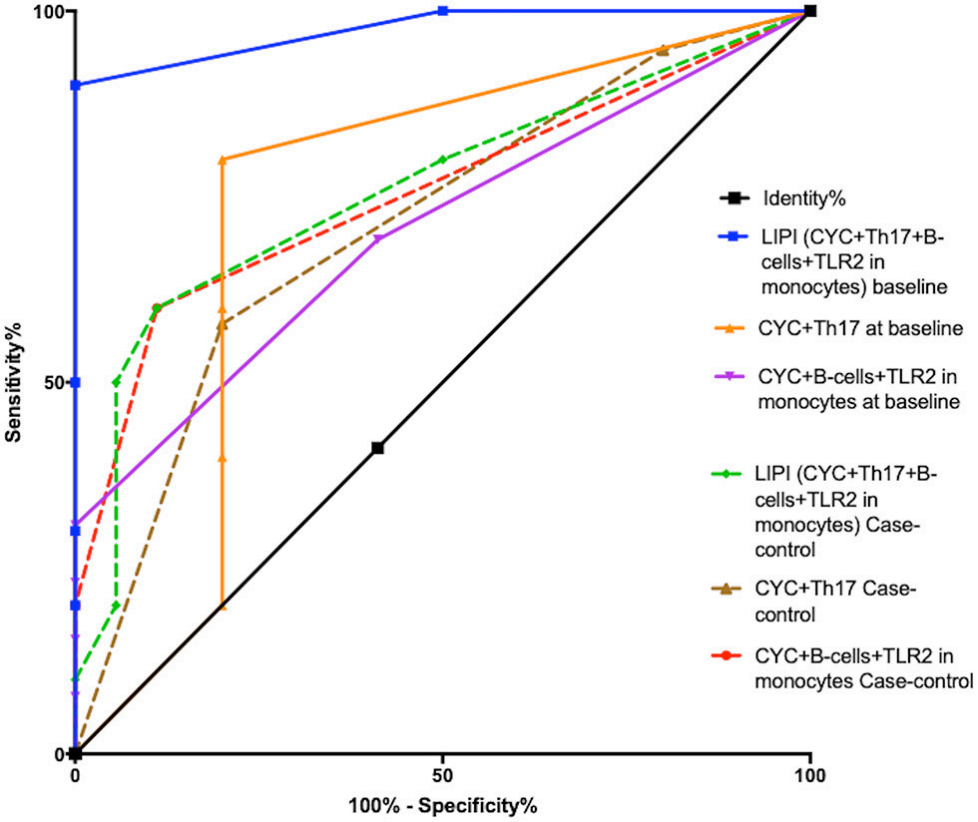
ROC curves showing the area under the curve (AUC) of the different sets of variables combinations using the baseline data set (continuous line) and in the cohort-nested case-control study (dotted lines).

As shown in Table 7 and Figure 4, the selected index was the one with the best AUC, which was the LIPI including the 4 variables at baseline. Although the AUC of the LIPI at baseline was not different from the AUC of the combination of cyclophosphamide use and absolute number of Th17, the LIPI showed a specificity of 100% and a higher negative LR than the former combination of variables with the baseline dataset. The AUC of the LIPI at baseline, was compared with the ROC test and was higher than the LIPI in the cohort nested case control study (*P* = 0.0025) and the combination of CYC+Th17 in the referred dataset (*P* = 0.035). Also, the latter combination of variables showed lower sensitivity as well as lower negative LR.

## Discussion

To our knowledge, this is the first prospective study to include the assessment of clinical and immunological parameters to develop an index for infection prediction in patients with SLE. Our most relevant finding is that the imbalance of Th17 and B cells numbers and a lower expression of TLR2 in monocytes along with cyclophosphamide use are the major risk factors for infection in SLE patients.

Infections are a burden in SLE, with a frequency ranging from 19.3 to 58.7%, and a mortality of 24.5% (6, 8), which agrees with our findings. Diverse risk factors for infections have been reported, such as age at diagnosis, Hispanic ethnicity, any use of glucocorticoids (≥10 mg/day), immunosuppressors, hospitalization by SLE, renal involvement and SLE damage index (SDI); while time on anti-malarials has been found as protective (8). Among these, we only corroborated the use of immunosuppressors (cyclophosphamide) as a major risk factor for infection, similar to the study by Bosch et al. (29). The higher risk of infection in patients receiving cyclophosphamide may be secondary to its cytotoxic effect, particularly upon proliferating lymphocytes (30). Furthermore, in animal models, cyclophosphamide use promotes translocation of intestinal bacteria (30), which could be another mechanism to explain its association with infection. In contrast with previous cohort studies, we did not find a protective effect of anti-malarials, probably because of the distinct follow-up time (8) and the methodological differences, since we performed a repeated measure analysis for every immunosuppressive drug and did not take into account the time on anti-malarials.

Interestingly, although we were not able to find association between the disease activity scales and the occurrence of infection, among our relevant findings are that patients with immunological signs of high disease activity including higher levels of Th17 cells, anti-dsDNA antibodies (31) and LDGs (27) were more prone to develop infections.

Two different subsets of Th17 cells have been identified in humans. The non-pathogenic subtypes whose main function is the defense against pathogens in the mucous membranes (32) and the pathogenic Th17, which are elevated in peripheral blood in lupus (31). In SLE, disease activity could be related to an imbalance between these subsets, with a predominance of the pathogenic Th17 and lower amounts of those cells devoted to defense toward infection.

Besides, we found that patients with lower levels of B-cells developed infection during the follow-up. In SLE, naïve B cells may be initially hyper-activated and produce higher levels of anti-dsDNA antibodies but this eventually leads to necroptosis (33), which would explain the lower B cell numbers in our study. Furthermore, in comparison to healthy controls, patients with SLE have lower frequency of BCR sequences with somatic hypermutations (SHM) (34). In animal models, the reduction in naïve B cells promotes *Cryptococcus* dissemination (35) and the lower biodiversity of BCR sequences with SHM in patients with SLE (34) may diminish the B cell repertoire to combat infections. Also, the lower pool of naïve B cells could lead to less class-switched memory B cells, which are known to be fundamental in the prevention of infections in other clinical conditions (36). The combination of a lower naïve B cell repertoire and diminished SHM may explain the increased risk of infection in SLE patients, although further studies are required to assess if different B cell subsets are involved in infection development in these patients.

We were not able to find the amount of NETs as a predictor of infection. However, we did find a differential protein cargo characterized by increasing amounts of LL-37 upon infection, probably as a defense mechanism. Besides, this antimicrobial peptide augments the expression of co-stimulatory molecules in dendritic cells (37) and promotes the production of type I IFN (38), which could be related to the association of SLE flares and infection. Indeed, the prospective design of this study allowed us to detect a disease flare in more than 50% of patients during infections.

Regarding TLR2, patients with SLE had a 56.6% lower expression in comparison to healthy controls. Although the regulating factors of the expression of TLR2 in SLE are unknown, glucocorticoids diminish its expression in keratinocytes and respiratory epithelial cells (39, 40). Previous studies had shown that patients with SLE had lower expression of TLR2 in monocytes in comparison to healthy controls (41), but this is the first study to describe it as a major risk factor for infection in lupus patients. Besides, the reduction of TLR2 has been acknowledged as a prognostic factor in infections, such as pneumonia (42) and its diminished expression may predispose SLE patients to infections in a similar way of genetic variants. The TLR2 Arg753Gln and T597C polymorphisms are related to Gram-positive septic shock, *S. aureus* and cytomegalovirus infections, as well as pulmonary and meningeal tuberculosis (43).

In summary, we propose that SLE patients have lower expression of TLR2 in monocytes. As a consequence, they have lower capacity to recognize and combat pathogens, since TLR2 is fundamental in the immune response against many bacterial, fungal, and viral infections. Besides, higher disease activity leads to Th17 expansion and B cell hyper-activation, with the consequent activation-induced necroptosis. The use of cyclophosphamide along with B cell necroptosis may promote a diminished pool of circulating B cells with a consequent limitation of the B cell repertoire to recognize, neutralize and combat pathogens.

Our data suggest that the combination of the use of cyclophosphamide with the compound measurement of B cells, Th17 cells, and TLR2 expression in monocytes is useful as an infection predictive index in SLE patients. All of the parameters included in the index are readily available using conventional flow cytometry, a technique that has demonstrated diagnostic utility as a compound measurement tool (44) as well as a cost-effective strategy (45) in many other diseases in the clinical setting, particularly at third level referral centers. Also, this is the first study to emphasize the pathogenic autoimmune response in SLE as a pivotal risk factor for infections, which could explain the concomitant occurrence of high disease activity and infections at lupus diagnosis even in the absence of immunosuppressive therapy. By alerting clinicians about patients who are more prone to develop infections, there could be a prompt and closer follow-up, with a multidisciplinary approach, including counsel by experts in infectious diseases. Additionally, since one of the most relevant prophylactic measures is the use of antibiotics, it is fundamental to detect patients with higher risk of infections to lower the generalized use of this preventive action in order to avoid the selection of resistant strains as it has been shown in other chronic diseases (46).

Our study has many limitations, including that our cohort is unicentric, solely composed by Hispanic patients with < 5 years since diagnosis and without any treatment with biologic anti-rheumatic disease drugs. Besides, our sample size is limited, and this could influence the lack of association between infection and some of the variables, such as the NET amount and the use of anti-malarials. Even though, a longer follow-up time would have been desirable, our study allowed us to find association of some of the variables with infection since the outcome developed early in the follow-up period. Furthermore, we should acknowledge that some of the variables included in the LIPI, as individual items, did show a small effect size for the prediction of infection. Nevertheless, the compound use of all these variables was able to increase their predictive capacity. Also, even though flow cytometry has gained access to the routine clinical setting, still it is not globally available and its cost-effectiveness should be addressed in future studies. Finally, this tool needs to be prospectively validated to identify a group of SLE patients with high risk of infection throughout the disease course to apply prophylactic measures regardless of immunosuppressive therapy and a closer follow-up.

## Author Contributions

DG-M participated in the conceptualization, funding acquisition, design, investigation process, project administration, supervision, data curation, formal analysis, validation, and visualization of the work. JT-R participated in the investigation process, data curation, formal analysis, validation and visualization of the work, and writing the original draft. NM-D participated in the formal analysis. AZ-D and MT-R provided software and resources for confocal mycroscopy. SM-P, RV-R, MA-L, RR-d-l-G, GJ-V, JM-C, and AB-V participated in data curation. JA-V participated in conceptualization, supervision, review, and editing the manuscript. AP-d-L participated in the supervision of the project.

### Conflict of Interest Statement

The authors declare that the research was conducted in the absence of any commercial or financial relationships that could be construed as a potential conflict of interest.

## References

[1] Pons-Estel GJ Ugarte-Gil MF Alarcon GS. Epidemiology of systemic lupus erythematosus. Expert Rev Clin Immunol. (2017) 13:799–814. 10.1080/1744666X.2017.1327352 28471259

[2] Tsokos GC Lo MS Costa Reis P Sullivan KE. New insights into the immunopathogenesis of systemic lupus erythematosus. Nat Rev Rheumatol. (2016) 12:716–30. 10.1038/nrrheum.2016.186 27872476

[3] Villanueva E Yalavarthi S Berthier CC Hodgin JB Khandpur R Lin AM. Netting neutrophils induce endothelial damage, infiltrate tissues, and expose immunostimulatory molecules in systemic lupus erythematosus. J Immunol. (2011) 187:538–52. 10.4049/jimmunol.1100450 21613614 PMC3119769

[4] Byrne JC Ni Gabhann J Lazzari E Mahony R Smith S Stacey K. Genetics of SLE: functional relevance for monocytes/macrophages in disease. Clin Dev Immunol. (2012) 2012:582352. 10.1155/2012/582352 23227085 PMC3511832

[5] Oliveira-Nascimento L Massari P Wetzler LM. The Role of TLR2 in Infection and Immunity. Front Immunol. (2012) 3:79. 10.3389/fimmu.2012.00079 22566960 PMC3342043

[6] Al-Rayes H Al-Swailem R Arfin M Sobki S Rizvi S Tariq M. Systemic lupus erythematosus and infections: a retrospective study in Saudis. Lupus (2007) 16:755–63. 10.1177/0961203307079943 17728372

[7] Lee YH Choi SJ Ji JD Song GG. Overall and cause-specific mortality in systemic lupus erythematosus: an updated meta-analysis. Lupus (2016) 25:727–34. 10.1177/0961203315627202 26811368

[8] Rua-Figueroa I Lopez-Longo J Galindo-Izquierdo M Calvo-Alen J Del Campo V Olive-Marques A. Incidence, associated factors and clinical impact of severe infections in a large, multicentric cohort of patients with systemic lupus erythematosus. Semin Arthritis Rheum. (2017) 47:38–45. 10.1016/j.semarthrit.2017.01.010 28259425

[9] Barber C Gold WL Fortin PR. Infections in the lupus patient: perspectives on prevention. Curr Opin Rheumatol. (2011) 23:358–65. 10.1097/BOR.0b013e3283476cd8 21532484

[10] Ng WL Chu CM Wu AK Cheng VC Yuen KY. Lymphopenia at presentation is associated with increased risk of infections in patients with systemic lupus erythematosus. QJM (2006) 99:37–47. 10.1093/qjmed/hci155 16371405

[11] Jeong SJ Choi H Lee HS Han SH Chin BS Baek JH. Incidence and risk factors of infection in a single cohort of 110 adults with systemic lupus erythematosus. Scand J Infect Dis. (2009) 41:268–74. 10.1080/00365540902744741 19172435

[12] Hochberg MC. Updating the American College of rheumatology revised criteria for the classification of systemic lupus erythematosus. Arthritis Rheum. (1997) 40:1725. 10.1002/art.1780400928 9324032

[13] Petri M Orbai AM Alarcon GS Gordon C Merrill JT Fortin PR. Derivation and validation of the systemic lupus international collaborating clinics classification criteria for systemic lupus erythematosus. Arthritis Rheum. (2012) 64:2677–86. 10.1002/art.34473 22553077 PMC3409311

[14] Heine G Sester U Sester M Scherberich JE Girndt M Kohler H. A shift in the Th(1)/Th(2) ratio accompanies the clinical remission of systemic lupus erythematosus in patients with end-stage renal disease. Nephrol Dial Transplant. (2002) 17:1790–4. 10.1093/ndt/17.10.1790 12270986

[15] Montoya-Ortiz G. Immunosenescence, aging, and systemic lupus erythematous. Autoimmune Dis. (2013) 2013:267078. 10.1155/2013/267078 24260712 PMC3821895

[16] Buyon JP Petri MA Kim MY Kalunian KC Grossman J Hahn BH. The effect of combined estrogen and progesterone hormone replacement therapy on disease activity in systemic lupus erythematosus: a randomized trial. Ann Intern Med. (2005) 142(12 Pt 1):953–62. 10.7326/0003-4819-142-12_Part_1-200506210-00004 15968009

[17] Isenberg DA Rahman A Allen E Farewell V Akil M Bruce IN. BILAG 2004. development and initial validation of an updated version of the British Isles Lupus Assessment Group's disease activity index for patients with systemic lupus erythematosus. Rheumatology (2005) 44:902–6. 10.1093/rheumatology/keh624 15814577

[18] Carmona-Rivera C Kaplan MJ. Induction and quantification of NETosis. Curr Protoc Immunol. (2016) 115:14.41.1–14. 10.1002/cpim.16 27801512

[19] Ibanez D Gladman DD Urowitz MB. Adjusted mean systemic lupus erythematosus disease activity index-2K is a predictor of outcome in SLE. J Rheumatol. (2005) 32:824–7. 15868616

[20] Buttgereit F da Silva JA Boers M Burmester GR Cutolo M Jacobs J. Standardised nomenclature for glucocorticoid dosages and glucocorticoid treatment regimens: current questions and tentative answers in rheumatology. Ann Rheum Dis. (2002) 61:718–22. 10.1136/ard.61.8.718 12117678 PMC1754188

[21] Moses T Holland PW. A comparison of statistical selection strategies for univariate and bivariate log-linear models. Br J Math Stat Psychol. (2010) 63(Pt 3):557–74. 10.1348/000711009X478580 20030964

[22] Dubberke ER Yan Y Reske KA Butler AM Doherty J Pham V. Development and validation of a clostridium difficile infection risk prediction model. Infect Control Hosp Epidemiol. (2011) 32:360–6. 10.1086/658944 21460487 PMC3649761

[23] Courtney PM Rozell JC Melnic CM Lee GC. Who should not undergo short stay hip and knee arthroplasty? Risk factors associated with major medical complications following primary total joint arthroplasty. J Arthroplasty. (2015) 30(Suppl. 9):1–4. 10.1016/j.arth.2015.01.056 26105617

[24] Harrell FE Jr. Lee KL Califf RM Pryor DB Rosati RA. Regression modelling strategies for improved prognostic prediction. Stat Med. (1984) 3:143–52. 10.1002/sim.4780030207 6463451

[25] Dolff S Bijl M Huitema MG Limburg PC Kallenberg CG Abdulahad WH. Disturbed Th1, Th2, Th17 and T(reg) balance in patients with systemic lupus erythematosus. Clin Immunol. (2011) 141:197–204. 10.1016/j.clim.2011.08.005 21920821

[26] Gomez-Martin D Diaz-Zamudio M Vanoye G Crispin JC Alcocer-Varela J. Quantitative and functional profiles of CD4+ lymphocyte subsets in systemic lupus erythematosus patients with lymphopenia. Clin Exp Immunol. (2011) 164:17–25. 10.1111/j.1365-2249.2010.04309.x 21235538 PMC3074213

[27] Denny MF Yalavarthi S Zhao W Thacker SG Anderson M Sandy AR. A distinct subset of proinflammatory neutrophils isolated from patients with systemic lupus erythematosus induces vascular damage and synthesizes type I IFNs. J Immunol. (2010) 184:3284–97. 10.4049/jimmunol.0902199 20164424 PMC2929645

[28] Talaat RM Mohamed SF Bassyouni IH Raouf AA. Th1/Th2/Th17/Treg cytokine imbalance in systemic lupus erythematosus (SLE) patients: correlation with disease activity. Cytokine (2015) 72:146–53. 10.1016/j.cyto.2014.12.027 25647269

[29] Bosch X Guilabert A Pallares L Cerveral R Ramos-Casals M Bove A. Infections in systemic lupus erythematosus: a prospective and controlled study of 110 patients. Lupus (2006) 15:584–9. 10.1177/0961203306071919 17080913

[30] Madondo MT Quinn M Plebanski M. Low dose cyclophosphamide: mechanisms of T cell modulation. Cancer Treat Rev. (2016) 42:3–9. 10.1016/j.ctrv.2015.11.005 26620820

[31] Wen Z Xu L Xu W Xiong S. Detection of dynamic frequencies of Th17 cells and their associations with clinical parameters in patients with systemic lupus erythematosus receiving standard therapy. Clin Rheumatol. (2014) 33:1451–8. 10.1007/s10067-014-2656-5 24810699

[32] Wacleche VS Landay A Routy JP Ancuta P. The Th17 lineage: from barrier surfaces homeostasis to autoimmunity, cancer, and HIV-1 pathogenesis. Viruses (2017) 9:E303. 10.3390/v9100303 29048384 PMC5691654

[33] Fan H Liu F Dong G Ren D Xu Y Dou J. Activation-induced necroptosis contributes to B-cell lymphopenia in active systemic lupus erythematosus. Cell Death Dis. (2014) 5:e1416. 10.1038/cddis.2014.375 25210799 PMC4225223

[34] Yin L Hou W Liu L Cai Y Wallet MA Gardner BP. IgM repertoire biodiversity is reduced in HIV-1 infection and systemic lupus erythematosus. Front Immunol. (2013) 4:373. 10.3389/fimmu.2013.00373 24298273 PMC3828670

[35] Dufaud C Rivera J Rohatgi S Pirofski LA. Naive B cells reduce fungal dissemination in *Cryptococcus neoformans* infected Rag1(−/−) mice. Virulence (2018) 9:173–84. 10.1080/21505594.2017.1370529 28837391 PMC5955176

[36] Lanio N Sarmiento E Gallego A Calahorra L Jaramillo M Navarro J. Alterations of naive and memory B-cell subsets are associated with risk of rejection and infection in heart recipients. Transpl Int. (2013) 26:800–12. 10.1111/tri.12131 23746145

[37] Hurtado P Peh CA. LL-37 promotes rapid sensing of CpG oligodeoxynucleotides by B lymphocytes and plasmacytoid dendritic cells. J Immunol. (2010) 184:1425–35. 10.4049/jimmunol.0902305 20042575

[38] Garcia-Romo GS Caielli S Vega B Connolly J Allantaz F Xu Z. Netting neutrophils are major inducers of type I IFN production in pediatric systemic lupus erythematosus. Sci Transl Med. (2011) 3:73ra20. 10.1126/scitranslmed.3001201 21389264 PMC3143837

[39] Su Q Pfalzgraff A Weindl G. Cell type-specific regulatory effects of glucocorticoids on cutaneous TLR2 expression and signalling. J Steroid Biochem Mol Biol. (2017) 171:201–8. 10.1016/j.jsbmb.2017.03.023 28377308

[40] Winder AA Wohlford-Lenane C Scheetz TE Nardy BN Manzel LJ Look DC. Differential effects of cytokines and corticosteroids on toll-like receptor 2 expression and activity in human airway epithelia. Respir Res. (2009) 10:96. 10.1186/1465-9921-10-96 19835594 PMC2772856

[41] Migita K Miyashita T Maeda Y Nakamura M Yatsuhashi H Kimura H. Toll-like receptor expression in lupus peripheral blood mononuclear cells. J Rheumatol. (2007) 34:493–500. 17295441

[42] Tang L Li Q Bai J Zhang H Lu Y Ma S. Severe pneumonia mortality in elderly patients is associated with downregulation of Toll-like receptors 2 and 4 on monocytes. Am J Med Sci. (2014) 347:34–41. 10.1097/MAJ.0b013e3182798583 23406892

[43] Noreen M Arshad M. Association of TLR1, TLR2, TLR4, TLR6, and TIRAP polymorphisms with disease susceptibility. Immunol Res. (2015) 62:234–52. 10.1007/s12026-015-8640-6 25784622

[44] Gibot S Bene MC Noel R Massin F Guy J Cravoisy A. Combination biomarkers to diagnose sepsis in the critically ill patient. Am J Respir Crit Care Med. (2012) 186:65–71. 10.1164/rccm.201201-0037OC 22538802

[45] Mason EF Morgan EA Pinkus GS Pozdnyakova O. Cost-effective approach to the diagnostic workup of B cell lymphoproliferative disorders via optimal integration of flow cytometric data. Int J Lab Hematol. (2017) 39:137–46. 10.1111/ijlh.12595 28133951

[46] Herath SC Normansell R Maisey S Poole P. Prophylactic antibiotic therapy for chronic obstructive pulmonary disease (COPD). Cochrane Database Syst Rev. (2018) 10:CD009764. 10.1002/14651858.CD009764.pub3 30376188 PMC6517028

